# Influence of the forest caterpillar hunter *Calosoma sycophanta* on the transmission of microsporidia in larvae of the gypsy moth *Lymantria dispar*

**DOI:** 10.1111/afe.12000

**Published:** 2013-01-22

**Authors:** Dörte Goertz, Gernot Hoch

**Affiliations:** * Department of Forest and Soil Sciences, BOKU–University of Natural Resources and Life SciencesHasenauerstraße 38, 1190 Vienna; † Department of Forest Protection, BFW–Federal Research Centre for ForestsSeckendorff-Gudent-Weg 8, 1131 Vienna, Austria

**Keywords:** Carabidae, host–pathogen interaction, Lymantriidae, *Nosema lymantriae*, predation, transmission, *Vairimorpha disparis*

## Abstract

1. The behaviour of predators can be an important factor in the transmission success of an insect pathogen. We studied how *Calosoma sycophanta* influences the interaction between its prey [*Lymantria dispar* (L.) (Lepidoptera, Lymantriidae)] and two microsporidian pathogens [*Nosema lymantriae* (Microsporidia, Nosematidae) and *Vairimorpha disparis* (Microsporidia, Burellenidae)] infecting the prey.
2. Using laboratory experiments, *C. sycophanta* was allowed to forage on infected and uninfected *L. dispar* larvae and to disseminate microsporidian spores when preying or afterwards with faeces.
3. The beetle disseminated spores of *N. lymantriae* and *V. disparis* when preying upon infected larvae, as well as after feeding on such prey. Between 45% and 69% of test larvae became infected when *C. sycophanta* was allowed to disseminate spores of either microsporidium.
4. Laboratory choice experiments showed that *C. sycophanta* did not discriminate between *Nosema*-infected and uninfected gypsy moth larvae*. Calosoma sycophanta* preferred *Vairimorpha*-infected over uninfected gypsy moth larvae and significantly influenced transmission.
5. When *C. sycophanta* was allowed to forage during the latent period on infected and uninfected larvae reared together on caged, potted oak saplings, the percentage of *V. disparis* infection among test larvae increased by more than 70%. The transmission of *N. lymantriae* was not affected significantly in these experiments.
6. Beetles never became infected with either microsporidian species after feeding on infected prey.
7. We conclude that the transmission of *N. lymantriae* is not affected. Because no *V. disparis* spores are released from living larvae, feeding on infected larvae might enhance transmission by reducing the time to death and therefore the latent period.

## Introduction

Arthropods and their natural enemies are embedded in a complex web of interactions. Two species can be directly involved in a trophic interaction, indirectly by competing for a joint resource or in a combination of both. Intraguild predation describes a situation when predators, parasitoids or pathogens share a host and are also engaged in a trophic interaction (Rosenheim *et al.*, 1995), possibly resulting in the release of the host from regulation and therefore the interruption of biological control (Meyling & Hajek, 2010). Other direct or indirect antagonistic interactions can arise when the population density of the host species is decreased to a point where transmission of a pathogen is interrupted, or when the nutritional quality of an infected host is altered, resulting in lower longevity, fecundity or the death of a parasitoid or predator (Hochberg *et al.*, 1990; Ruberson *et al.*, 1991; Brooks, 1993; Rosenheim *et al.*, 1995; Sajap *et al.*, 1999). By contrast, benefits for at least one of the interacting enemies of a host species may arise when parasitism enhances the susceptibility of the host to predation or when the action of a predator or parasitoid enhances the transmission of a pathogen by defecation of environmental stable life stages or vectoring the pathogen to susceptible hosts (Carpinera & Barbosa, 1975; Brooks, 1993; Roy & Pell, 2000; Castillejos *et al.*, 2001; Down *et al.*, 2004).

The gypsy moth *Lymantria dispar* (L.) (Lepidoptera, Lymantriidae) is a well known species that is native to Europe and Asia and, after introduction, in North America. Its larvae are polyphagous but feed preferentially on oak trees in Central Europe. Larvae are present from late April to late June/early July (Wellenstein & Schwenke, 1978). *Lymantria dispar* itself is also host for several microsporidian pathogens, some of which are considered for inoculative releases in North American *L. dispar* populations (McManus & Solter, 2003). *Vairimorpha disparis* (Microsporidia, Burellenidae) and *Nosema lymantriae* (Microsporidia, Nosematidae) infect gypsy moth larvae, when spores are ingested with food. An initial developmental cycle starts in the midgut and leads eventually to the formation of primary spores. These spores spread to the target tissues of the host larvae and, after a secondary developmental cycle, environmentally stable spores are formed. V*airimorpha disparis* is a fat body parasite. Environmental spores produced for the transmission between host larvae can be found in the fat body after 7 days; the fat body is filled with spores after 10 days and an infected larva dies a mean of 4 weeks after infection under laboratory conditions (Goertz & Hoch, 2008a). Horizontal transmission takes place when spores are released from the decomposing cadaver. *Nosema lymantriae* is a systemic parasite that infects the silk glands, the fat body, the gonads and the Malpighian tubules of a host larva. Of the infected larvae, 95% will die 4 weeks after infection. Horizontal transmission of this species starts after the end of the latent period when spores are released with faeces approximately 2 weeks after infection, and also continues after the death of the host larva when its cadaver decomposes (Goertz & Hoch, 2008a). *Nosema lymantriae* is also transovarially transmitted by infected females to its progeny (Goertz & Hoch, 2008b).

*Calosoma sycophanta* L. (Coleoptera, Carabidae) is a voracious predatory beetle that is known to occur in high abundance during outbreaks of *L. dispar* (Burgess & Crossman, 1929; Weseloh, 1985a; Hoch *et al.*, 2006). This beetle is known to feed on many forest pest Lepidoptera, such as the pine-tree lappet moth *Dendrolimus pini* L., the nun moth *Lymantria monacha* L., the pine processionary moth *Thaumetopoea pityocampa* Dennis and Schiffermüller and the oak processionary moth *T. processionae* L. (Escherich, 1942). Both, larvae and adults of *C. sycophanta* forage for larval and pupal stages of their prey, which is attacked vigorously and killed eventually. *Calosoma sycophanta* then feed on body fluids or substances and consume most of their prey. Burgess and Crossman (1929) report that a pair of adult beetles and their progeny can kill more than 6000 *L. dispar* larvae and pupae within one season. This beetle was introduced to the U.S.A. at the beginning of the last century for the control of *L. dispar* and is now a well established predator. *Calosoma sycophanta* pupae have also been mass released for biological control of *T. pityocampa* in pine forest in different regions of Turkey (Kanat & Toprak, 2005; Kanat & Zbolat, 2006; Kanat & Mol, 2008).

In gypsy moth areas, adult beetles emerge from their hibernating chambers in the soil at the beginning of June and feed on *L. dispar* larvae. Female beetles lay a mean of 100 eggs (Burgess & Crossman, 1929) in small batches into the soil (Schwerdtfeger, 1981). Larvae hatch and start to search and prey preferentially on gypsy moth pupae (Weseloh, 1988). Beetles and larvae foraging in *L. dispar* populations come into contact with other natural enemies and pathogens of their prey and interact with them.

In the present study, we tested whether a predator such as *C. sycophanta* can influence the interaction between *L. dispar* and its microsporidian pathogens, and also whether *C. sycophanta* itself is affected by the microsporidian pathogens. Moreover, in the laboratory, we tested whether the beetle discriminates between infected and uninfected prey and whether it is able to disseminate spores when preying or afterwards with its faeces. In experiments with caged, potted oak plants we quantified the influence of preying *C. sycophanta* on horizontal transmission of *N. lymantriae* or *V. disparis*.

## Materials and methods

### Insects and pathogens

*Lymantria dispar* larvae of the New Jersey Standard Strain served as the host insect for all experiments outlined in the present study and as prey for *C. sycophanta* during the standard rearing procedure before and after the experiments. Egg masses were obtained from the USDA-APHIS Otis Method Development Center (Otis, Massachusetts). Regularly, larvae of *L. dispar* obtained from these egg masses were microscopically examined and confirmed to be free of microsporidian infections. Unless otherwise indicated, larvae were reared at a temperature of 24 : 18 °C (light : dark) under an LD 16 : 8 h photocycle individually or in small groups in plastic cups (50 or 250 mL, respectively) on a wheat germ diet (Bell *et al.*, 1981).

*Calosoma sycophanta* beetles were obtained from the laboratory of Dr M. Kanat (Kahramanmaraş Sütçüİmam University, Turkey) 2008 or from field collections in Bavaria (Germany) in 2009 and reared as described by Weseloh (1985a, 1993) and Kanat and Zbolat (2006). Adult beetles were reared in plastic cups (volume 250 mL, diameter 9.5 cm, height 5.5 cm) filled with potting soil moistened with distilled water under an LD 16 : 8 h photocycle and at 24 °C. Adult beetles were fed daily with four to five fourth- or fifth-instar *L. dispar* larvae. Before the first set-up of experiments, several beetles were dissected and checked for microsporidian infections. All *C. sycophanta* beetles were used only once for one experiment to avoid cross contamination and had no contact with infected *L. dispar* larvae before the experiment. At the end of the experiments, all tested *C. sycophanta* beetles were microscopically examined for a microsporidian infection.

Two different microsporidian species were used for all experiments. *Nosema lymantriae* (Isolate No. 1996-A) was originally isolated from silk glands of *L. dispar* larvae that were collected in 1996 near Levishte, Bulgaria. *Vairimorpha disparis* (Isolate No. 1995-C) was isolated from fat bodies of *L. dispar* larvae, collected in 1995 near Rupite, Bulgaria. Both microsporidian species are stored in the microsporidia germ-plasma collection of the Illinois Natural History Survey, Urbana-Champaign, Illinois (laboratory of Dr L. F. Solter) from which they were originally obtained. Microsporidian spores for the experiments were produced in *L. dispar* larvae (Goertz & Hoch, 2008a) and harvested from infected tissues 16 days post-inoculation (dpi). They were cleaned by filtration through cellulose tissue and centrifugation. Spore suspensions in distilled water were mixed 1 : 1 with glycerol and stored in liquid nitrogen (Maddox & Solter, 1996) for no longer than 3 months until used in the experiments.

### Microsporidian infections

Newly-moulted third-instar *L. dispar* larvae were infected with microsporidia in accordance with the procedure described by Bauer *et al.* (1998) and Goertz and Hoch (2008a). *Lymantria dispar* larvae were fed individually 1 µL of spore suspension with a concentration of 1 × 10^3^ spores/µL of *N. lymantriae* or *V. disparis* on a diet cube (2 mm^3^). This dosage causes 100% infection in *L. dispar* larvae (Goertz & Hoch, 2008a). Spore suspensions were counted in a Neubauer haemacytometer and adjusted to the required concentration with distilled water. Control larvae were inoculated with distilled water. Only larvae that ingested the entire diet cube within 24 h were used for the experiments.

At the end of each experiment, all larvae were checked for a microsporidian infection. Cross sections of each larva were made with fine scissors; the preparations were microscopically examined using phase contrast microscopy with a 20–40-fold magnification.

### *Discrimination between infected and uninfected prey by* C. sycophanta

Choice experiments were conducted to determine whether *C. sycophanta* beetles discriminate between infected and uninfected prey. Five uninfected and five infected *L. dispar* larvae of the same size and age (at 16 and 17 dpi, fourth or fifth instar) were placed into each of six rearing cages (height 29 cm, diameter 20 cm) for each treatment (*V. disparis*, *N. lymantriae*) onto watered oak foliage bouquets of *Quercus petraea*. At this point, numerous microsporidian spores were present the target tissues and infected larvae were severely affected by the infection. All larvae were allowed to acclimate for 2 h. Subsequently, one *C. sycophanta* beetle (Turkey) that had been starved for 24 h was placed in each cage and allowed to prey on the *L. dispar* larvae for 24 h. Six beetles were used for each treatment. The experiment was performed twice. No *L. dispar* larvae were replaced during the 24 h of their exposure period. The next day, all remaining live larvae and larval cadavers were collected. The number of larval cadavers was counted. Every live larva was checked microscopically for the status of infection and the number of consumed infected or uninfected larvae was calculated.

To test for preference of one prey type, log ratios (LR) were calculated as described by Roy *et al.* (2008):


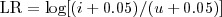


where *i* is the number of consumed infected larvae and *u* is the number of consumed uninfected larvae. When no preference occurs LR = 0, LR < 0 indicates a preference for uninfected larvae and LR > 0 indicates a preference for infected larvae. A one-sample *t*-test was calculated to test for significant deviation of LR from 0. All data were analyzed using SPSS, version 15.0.0 (SPSS Inc., Chicago, Illinois).

### *Spore dissemination when preying on infected* L. dispar *larvae*

This experiment was carried out to determine whether *C. sycophanta* disseminates microsporidian spores that originate directly from the larval body when the beetle is feeding on infected *L. dispar* larvae. *Calosoma sycophanta* beetles (Turkey and Germany) were starved for 24 h before use in the experiment.

Five larvae infected with *N. lymantriae*, were placed onto watered oak foliage bouquets (*Q. petraea*) in each of 12 rearing cages (height 29 cm, diameter 20 cm) at 16 dpi and allowed to acclimate for 2 h. Five larvae infected with *V. disparis* (16 dpi) were placed into each of another 12 cages. One *C. sycophanta* beetle per cage was allowed to prey on the infected larvae for 24 h in six cages of each pathogen treatment. The *Nosema*- and *Vairimorpha*-infected larvae in the other 2 × 6 cages remained undisturbed for 24 h. The next day, all beetles, remaining *L. dispar* larvae, larval cadavers and faeces of both, larvae and beetle, were removed from the cages by re-collecting beetles, larvae, the larval cadavers and faecal pellets and by disinfecting places where *C. sycophanta* defecated with 70% ethanol. The number of larval cadavers was recorded. Then, 10 uninfected third-instar *L. dispar* (= test larvae) were placed onto the oak foliage bouquets for a period of 3 days. After the exposure period, all test larvae were removed from the cages and reared individually on diet for 20 days to allow any acquired infection to develop. All test larvae were dissected at the end of the experiment. The experiment was performed twice for a total of 12 replicates.

A Kruskal–Wallis *H*-test followed by pairwise Tukey–Kramer tests was applied to examine differences between treatments because the data did not follow a normal distribution according to Kolmogorov–Smirnoff tests.

### *Spore dissemination through* C. sycophanta *faeces*

To test for the dissemination of microsporidian spores through faeces of *C. sycophanta* beetles, twelve starved adult *C. sycophanta* (Turkey) were fed with five infectious *L. dispar* larvae at 16 dpi inoculated with either *N. lymantriae* or *V. disparis*. The next day, the beetles were rinsed with 70% ethanol and water to remove microsporidian spores that might have adhered to their cuticula and placed individually into each of 12 cages (height 29 cm, diameter 20 cm) containing oak foliage bouquets (*Q. petraea*) and five uninfected *L. dispar* larvae that had been introduced 2 h earlier. After 24 h, all beetles, remaining live larvae, larval cadavers and larval faeces were removed by re-collecting beetles, larvae, the larval cadavers and faecal pellets with 70% ethanol and ten newly-moulted uninfected third instars (= test larvae) were placed onto the oak bouquets and allowed to feed for 3 days. After the exposure period, all test larvae were reared individually on diet for 20 days to allow any acquired infection to develop. The larvae were then dissected and examined for a microsporidian infection. The experiment was performed twice.

### *Influence of* C. sycophanta *on horizontal transmission of microsporidia*

This experiment was conducted to test whether adult *C. sycophanta* can influence the transmission of *N. lymantriae* or *V. disparis* under more natural conditions and before infected *L. dispar* larvae become infectious or, in case of *N. lymantriae*, shortly after the beginning of the infectious period. The experiment was performed twice with three and four replicates for each treatment and microsporidian species in 2008 and 2009, respectively. *Calosoma sycophanta* beetles used for this experiment originated from Turkey (2008) and Germany (2009) and were starved for 24 h before use in the experiment.

Newly-moulted *L. dispar* third instars were individually inoculated with either microsporidian species and reared first on wheat germ diet until 5 dpi and then on oak foliage. They were permanently marked by clipping one proleg 2 dpi; this procedure does not lead to an increased mortality and does not reduce the mobility of the larvae (Weseloh, 1985b; Hoch *et al.*, 2008).

Nine infected and marked *L. dispar* larvae at 10 dpi and 21 uninfected newly-moulted third instars (= test larvae) were placed onto potted 2-year-old and 1-m high oak saplings (*Q. petraea*). The larvae were allowed to feed together either for 5 days (10–15 dpi) during the latent period of the infected larvae and microsporidian infection (Table 1) or for 10 days (10–20 dpi) during the latent and infectious period of the infected larvae. *Calosoma sycophanta* beetles, one per cage, were allowed to forage on infected or uninfected larvae in two-thirds of the cages for 48 h either during the latent period or just after the beginning of the infectious period (Table 1). Larvae of the remaining cages were undisturbed. Thirty uninfected newly-moulted third instars were placed onto additional oak plants and held for 5 and 10 days as negative controls. All potted oak plants were placed into mesh bags (1-mm^2^ mesh) that were fixed to the plant pot with double-sided adhesive tape to prevent predation or parasitization by other insects. After the exposure period, all test larvae were reared individually on diet for 20 days to allow any acquired infection to develop. All test larvae were dissected at the end of the experiment to determine whether they were infected.

**Table 1:** Experimental set-up for the horizontal transmission experiment showing the tested treatments and control groups

Treatment					
			
Microsporidian species	Exposure of *Lymantria dispar* larvae (dpi)	Predation by *Calosoma sycophanta* (dpi)	Replicates	State of microsporidian infection	Description
Either	10–15	None	7	Latent period	No-beetle control
*Nosema lymantriae* or *Vairimorpha disparis*	10–15	11–13	7	Latent period	Early predation
	10–20	None	7	Latent and infectious period	No-beetle control
	10–20	11–13	7	Latent and infectious period	Early predation
	10–20	16–18	7	Latent and infectious period	Late predation
Controls	10–15	None	2	–	Control for contamination with either
	10–20	None	2	–	microsporidian species

Both microsporidian species were tested. Among the treatments, the exposure period of *L. dispar* larvae and the time when *C. sycophanta* was allowed to forage on infected and uninfected larvae were varied. The predation period of *C. sycophanta* and the number of trials as well as the status of microsporidian development in infected larvae is also shown. dpi, days post-inoculation.

The datasets of multiple dependent scale variables in this experiment were analyzed by multivariate analysis of variance followed by a post-hoc test (least significant difference) using the general linear model multivariate procedure of SPSS, with the exposure period of larvae and the predation period of beetles as factors and the percentage of test larvae that became infected and the recovery rates of inoculated and test larvae as dependent variables. Frequency datasets were arcsin(^2^√*p*) transformed and Box's *M* was used to test the null hypothesis that the observed covariance matrices of the dependent variables were equal across groups.

## Results

### *Discrimination between infected and uninfected prey by* C. sycophanta

None of the beetles consumed more than four of the five offered larvae of either prey type. *Calosoma sycophanta* beetles showed a significant preference for *Vairimorpha*-infected over uninfected larvae (one-sample *t*-test, *t* = 1.993, *P* < 0.05; Table 2). On average, they consumed approximately twice as many infected as uninfected larvae. When *C. sycophanta* adults were offered the choice between uninfected and *Nosema*-infected larvae, they consumed approximately one larva of each prey type and did not show any significant preference for either prey type (one-sample *t*-test, *t* = 0.296, *P* > 0.05; Table 2). None of the tested beetles became infected with either microsporidian species.

**Table 2:** Number of larvae (mean ± SD; maximum) consumed by an individual beetle during one test, log ratio (LR) (mean ± SD) describing preference and the results of a one-sample *t*-test

Prey	Mean ± SD	Maximum	Log ratio, mean ± SD	*t*	d.f.
*Vairimorpha* disparis					
Infected	0.9 ± 0.8	2	0.575 ± 1.000	1.993*	11
Uninfected	0.4 ± 0.8	2			
*Nosema lymantriae*					
Infected	1.1 ± 1.4	4	−0.120 ± 1.340	0.296	11
Uninfected	1.1 ± 1.0	2			

* LR is significantly different from 0 at *P*≤ 0.05.

### *Spore dissemination when preying on infected* L. dispar *larvae*

**Figure 1:**
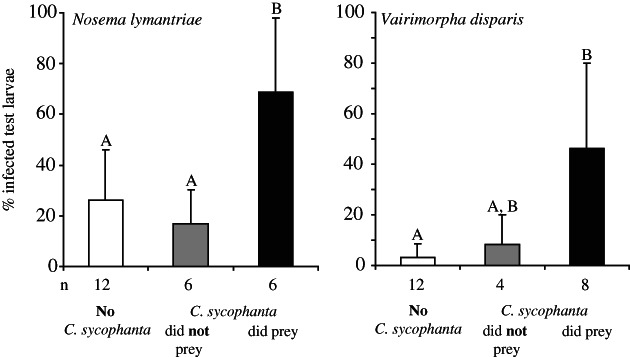
Percentage infection (mean ± SE) of test larvae feeding on foliage that was possibly contaminated with spores of *Nosema lymantriae* or *Vairimorpha disparis* after predation of *Calosoma sycophanta* on microsporidia-infected *Lymantria dispar* larvae. Uppercase letters above bars indicate significant differences between groups (Kruskal–Wallis *H*-test followed by pairwise Tukey–Kramer tests).

Transmission of both microsporidian species was enhanced when *C. sycophanta* beetles preyed on infected *L. dispar* larvae (Fig. 1). The proportion of third-instar test larvae that became infected with microsporidia increased significantly by 2.6-fold (*N. lymantriae*, *H*-test: *χ*^2^ = 9.75, d.f. = 2, *P* = 0.008) and 14.9-fold (*V. disparis*, *H*-test: *χ*^2^ = 6.72, d.f. = 2, *P* = 0.035). When *C. sycophanta* beetles were present in the cages but did not consume any *L. dispar* larvae, the percentage infection of test larvae did not differ significantly from cages with no beetles present. Neither *N. lymantriae*, nor *V. disparis* was transmitted to *C. sycophanta* beetles.

### *Spore dissemination through* C. sycophanta *faeces*

*Calosoma sycophanta* consumed between one and two *Nosema*-infected larvae before the experimental period. Of the *L. dispar* test larvae, 68.7% ± 11.9% became infected when they fed on foliage contaminated by the faeces of *C. sycophanta* in the cages. Between one and four *Vairimorpha*-infected larvae were consumed by *C. sycophanta* before the experimental period. When allowed to feed on potentially contaminated foliage, 46.3% ± 23.9% of the test larvae became infected. No microsporidian infection was found in *C. sycophanta* beetles used in this experiment.

### *Influence of* C. sycophanta *on horizontal transmission of microsporidia*

#### Nosema lymantriae

**Figure 2:**
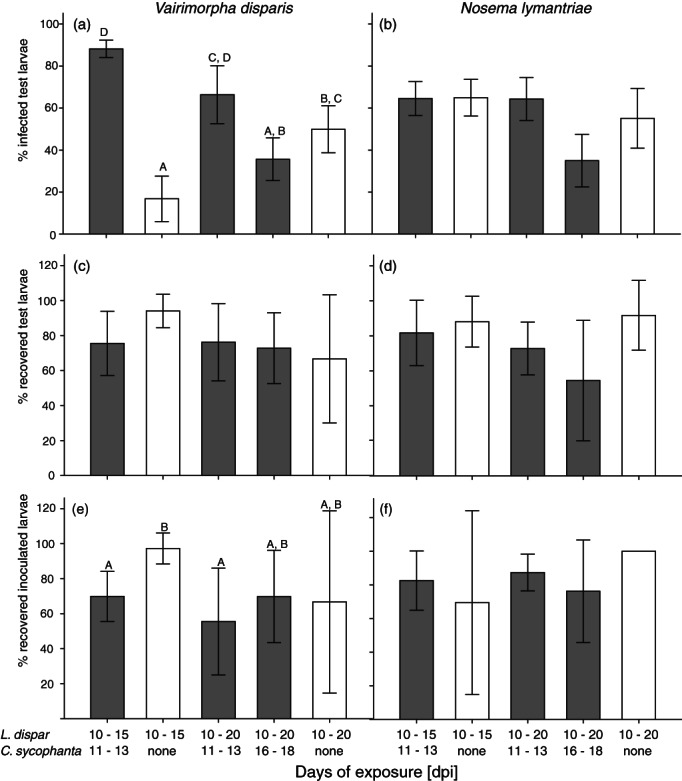
Influence of *Calosoma sycophanta* on the percentage infection of *Lymantria dispar* test larvae (a, b), their recovery rate (c, d) and the proportion of recovered inoculated larvae (e, f) (mean ± SE). White bars represent the ‘no-beetle’ control with short [10–15 days post-inoculation (dpi)] and long (10–20 dpi) larval exposure periods; grey bars represent treatments with short (10–15 dpi) and long (10–20 dpi) exposure periods of *L. dispar* and with *C. sycophanta* beetles present either at 11–13 or 16–18 dpi. Uppercase letters above bars indicate significant differences between groups (multivariate analysis of variance, least significant difference); absence of letters indicates that differences were not significant.

On average, 90.5% and 80.0% of the inoculated larvae were recovered from potted oak plants at the end of the exposure period in 2008 and 2009, respectively. Of the test larvae, on average, 84.7% and 74.8% were re-collected in 2008 and 2009. This proportion varied between 54.5% and 93.8% in all treatments and was not influenced by the duration of the exposure period or the presence of *C. sycophanta* (Fig. 2 and Table 3). The transmission of *N. lymantriae* was affected neither by the presence of the predatory beetle, nor by the length of the exposure period. A significant interaction between ‘presence of *C. sycophanta*’ and ‘length of exposure period’ was not found. On average, between 34.9% and 64.9% of the test larvae became infected with *N. lymantriae*. None of the tested beetles became infected with *N. lymantriae*.

**Table 3:** Results of a multivariate analysis of variance for the experiments with *Nosema lymantriae* with an ‘exposure period’ of *Lymantria dispar* larvae in the cages (10–15 and 10–20 days post-inoculation), ‘*Calosoma sycophanta*’ (no beetle present, beetle present early and beetle present late) and ‘year’ (2008 and 2009) as factors and the percentage infection of test larvae, the percentage of recovered test and the percentage of inoculated larvae (all transformed) as dependent variables (d.f. = 18, Box's' *M* = 36.67, *P* = 0.406)

Factor	Dependent variable	Mean square	d.f.	*F*	*P*	Partial *η*^2^
Exposure period	Percentage infected test larvae	0.061	1	0.46	0.50	0.02
	Recovered test larvae	0.092	1	1.15	0.30	0.05
	Recovered inoculated larvae	0.028	1	0.25	0.62	0.01
*Calosoma sycophanta*	Percentage infected test larvae	0.459	1	3.49	0.07	0.13
	Recovered test larvae	0.101	1	1.26	0.27	0.05
	Recovered inoculated larvae	0.011	1	0.10	0.27	0.00
Year	Percentage infected test larvae	0.022	1	0.17	0.69	0.01
	Recovered test larvae	0.339	1	4.22	0.051	0.15
	Recovered inoculated larvae	0.214	1	1.99	0.17	0.08

#### Vairimorpha disparis

On average, 72.2% and 73.9% of the inoculated larvae were re-collected in 2008 and 2009, respectively. Of the test larvae, 79.0% and 78.1% were re-collected in 2008 and 2009, respectively. The percentage of recovery was not significantly influenced by the presence of *C. sycophanta*; the duration of exposure period significantly affected the recovery of incoculated larvae (Fig. 2 and Table 4). The transmission of *V. disparis* was influenced by the presence of *C. sycophanta* (Fig. 2 and Table 4). When *C. sycophanta* was allowed to forage before the end of the latent period (11–13 dpi), a higher percentage of susceptible test larvae became infected. None of the tested *C. sycophanta* beetles became infected with *V. disparis*.

**Table 4:** Results of a multivariate analysis of variance for the experiments with *Vairimorpha disparis* with ‘exposure period’ of *Lymantria dispar* larvae in the cages (10–15 and 10–20 days post-inoculation), ‘*Calosoma sycophanta*’ (no beetle present, beetle present early and beetle present late) and ‘year’ (2008 and 2009) as factors and the percentage infection of test larvae and the percentage of recovered test and inoculated larvae as dependent variables (d.f. = 24, Box's *M* = 23.19, *P* = 0.262)

Factor	Dependent variable	Mean square	d.f.	*F*	*P*	Partial *η*^2^
Exposure period	Percentage infected test larvae	1708.575	1	3.72	0.07	0.13
	Recovered test larvae	665.390	1	2.24	0.15	0.09
	Recovered inoculated larvae	2788.900	1	5.46	0.03	0.19
*Calosoma sycophanta*	Percentage infected test larvae	7028.478	1	15.29	0.001	3.89
	Recovered test larvae	47.715	1	0.16	0.69	0.01
	Recovered inoculated larvae	1492.613	1	2.92	0.10	0.11
Year	Percentage infected test larvae	60.184	1	0.13	0.72	0.01
	Recovered test larvae	5.080	1	0.02	0.90	0.001
	Recovered inoculated larvae	10.308	1	0.02	0.89	0.001

None of the test larvae exposed on oak plants without inoculated larvae or beetles present (negative controls) acquired a microsporidian infection.

None of the larvae of the negative controls became infected with *N. lymantriae* or *V. disparis*.

## Discussion

Although there are a number of studies that consider the interactions of microsporidian pathogens and parasitoids sharing a host (Brooks, 1993), very few studies have investigated the interactions between predators and microsporidia feeding on the same host species (Van Essen & Anthony, 1976; Down *et al.*, 2004). When *Nosema* (= *Anncaliia*) *algerae* was fed to predators of mosquitoes, one of the nine tested predatory species, *Notonecta undulate* (Hemiptera), became infected (Van Essen & Anthony, 1976). In a more extensive study, Down *et al.* (2004) reported that the spined soldier bug *Podisus maculiventris* disseminated spores of *Vairimorpha necatrix* on tomato plants with their faeces, thereby causing the reduced survival of *Lacanobia oleracea* and *Spodoptera littoralis* larvae by 61% and 75%, respectively. They concluded that *P. maculiventris* is a very effective disseminator of *V. necatrix* and might be useful for introducing this pathogen into a pest population and for enhancing the effectiveness of this biological control agent. The present study shows that a predator such as *C. sycophanta* can influence the host–pathogen interaction of *L. dispar* and its microsporidia at a small scale and that *C. sycophanta* itself is not infected by the microsporidian pathogens.

Infection of the predator when feeding on infected prey can be one important nontarget effect in biological control (Rosenheim *et al.*, 1995). In our experiments, *C. sycophanta* did not become infected with either *N. lymantriae* or *V. disparis* after feeding on infected prey. An infected larval *L. dispar* cadaver contains more than 4 × 10^9^ microsporidian spores (Goertz & Hoch, 2008a). Hence, we assume that *C. sycophanta* ingests more than 1 × 10^9^ spores when feeding on one larva, which is approximately the 10^6^-fold lethal dosage for the *L. dispar* larvae. One basis for this resistance may be the difference in pH in the midguts, which is higher in lepidopteran larvae than in other insects (Chapman, 1998). The germination of microsporidian spores depends strongly on several stimuli such as pH or ion concentrations (Undeen, 1990; Cali & Takvorian, 1999). Therefore, it is possible that the failure of both microsporidian species to infect *C. sycophanta* is the result of unfavorable conditions in the midgut of the predatory beetle, as previously suggested by Down *et al.* (2004) to explain the failure of *Vairimorpha necatrix* spores to infect the predatory bug *Podisus maculiventris*. We conclude that *C. sycophanta* is not infected after consuming either *N. lymantriae* or *V. disparis*-infected larvae.

Approximately 60% of the test larvae became infected after feeding on foliage that had been contaminated by faeces of *C. sycophanta* that fed on infected prey. This indicates that spores of both microsporidia remained viable after passing through the beetle's digestive tract. The cleaning procedure of the beetles in the experiments and the high percentage of infected test larvae make it unlikely that spores adhering to the beetle's surface were the source of infections acquired by the test larvae. In an experiment using the same arena, Goertz and Hoch (2011) measured a transmission of 57% resulting from the contamination of foliage with spore-laden faeces from *Nosema*-infected larvae. Transmission originating from cadavers containing spores of either *N. lymantriae* or *V. disparis* was approximately 15%. Thus, the proportion of larvae that became infected after feeding on foliage contaminated by *C. sycophanta* in the present study is in the same range or even higher. Therefore, *C. sycophanta* might cause the same level of transmission of microsporidian pathogens as the infectious larva itself or might enhance the transmission of *V. disparis* when it selectively preys on infected larvae. Furthermore, *C. sycophanta* may contribute to the dispersal of microsporidian pathogens in the habitat of *L. dispar*. It has been shown that viable fungal spores or viral occlusion bodies can be transmitted and dispersed by predators or parasitoids into new habitats (Entwistle *et al.*, 1977; Lee & Fuxa, 2000; Roy & Pell, 2000). Carpinera and Barbosa (1975) found that faecal samples of field collected *C. sycophanta* contained, on average, 3.1 × 10^9^ viral polyhedra/cm^3^. Because *C. sycophanta* is actively searching for its prey, such dispersal of the microsporidium in the host population would take place in a targeted way, as demonstrated for the lady beetle *Coccinella septempunctata* by Roy *et al.* (2001).

Several studies (Müller, 1983; Furlong & Pell, 1996; Roy & Pell, 2000) reported that the presence of a predator can result in disturbance, dramatic escape response by its prey and, consequently, a higher transmission of fungal pathogens. The results of the present study do not indicate such an interaction. When *C. sycophanta* was present in a cage but did not consume larvae, the resulting percentage infection of test larvae was not significantly higher compared with the no-beetle control (Fig. 1). The mere presence of the beetle does not appear to result in an increased movement of infected larvae, a higher contamination of the larval environment and therefore a higher transmission of microsporidia. By contrast, the act of killing and consuming infected larvae supposedly contributes to a significant contamination of the environment, as indicated by the significantly increased transmission in our corresponding experiments.

The results of the present study show that *L. dispar* larvae infected with *N. lymantriae* are not preferred or avoided over uninfected larvae when given the choice. The transmission of *N. lymantriae* was not affected in cages with potted oak plants when *C. sycophanta* was allowed to prey on infected or uninfected *L. dispar* larvae. Spores of *N. lymantriae* are released from feeding larvae in faeces (Goertz & Hoch, 2008a) contributing to its successful transmission. This leads to the conclusion that, irrespective of the presence of *C. sycophanta*, a sufficient number of *Nosema* spores are released for the successful transmission of this microsporidian species.

*Nosema lymantriae* is horizontally and vertically transmitted in *L. dispar* populations (Goertz & Hoch, 2008a, b). Infected females may transmit this microsporidium to their progeny leading to the early death of these infected larvae and initiating horizontal transmission cycles among young or middle-aged *L. dispar* larvae (Hoch & Goertz, 2009). These larvae further contribute to the spread of *N. lymantriae* by releasing spores with faeces and after death. *Calosoma sycophanta* may additionally contribute to the spread and increase the likelihood of the vertical transmission of *N. lymantriae*. After feeding on older, infected *L. dispar* larvae, *C. sycophanta* will spread *N. lymantriae* spores that may cause additional infections in larvae just before pupation. The acquired infections will not result in the death of the infected specimens but, instead, vertical transmission for those female larvae that became infected. *Vairimorpha disparis* is transmitted from the decomposing cadaver after host death, which occurs approximately 4 weeks after ingesting infective spores (Goertz & Hoch, 2008a). High numbers of environmental spores are already present in the fat bodies of infected larvae approximately 10 days after infection (Goertz & Hoch, 2008a). Therefore, *C. sycophanta* may contribute to the horizontal transmission and spread of *V. disparis* when feeding preferentially on infected larvae during the whole larval stage of *L. dispar* and by shortening the latent period.

In summary, we conclude that *C. sycophanta* is not a host for *N. lymantriae* or *V. disparis* but can disperse viable spores either of the species within *L. dispar* populations. We do not expect a negative effect on both microsporidian species. *Calosoma sycophanta* did not cause a negative effect on the transmission of *N. lymantriae* by selectively preying and removing *Nosema*-infected *L. dispar* larvae. Moreover, *C. sycophanta* might enhance the transmission of the fat body parasite *V. disparis* when it preys upon infected larvae by reducing the latent period of the infection. Our small-scale experiments indicate that the transmission success of *N. lymantriae* and *V. disparis* was affected differently. The difference is likely a result of the different pathways for horizontal transmission: *N. lymantriae* spores are released from feeding larvae through faeces, whereas the release of *V. disparis* spores usually begins after the death of the host. Thus, predation by *C. sycophanta* apparently led to an earlier dissemination of *V. disparis* spores that are produced in the host's fat body. Targeted dispersal of inoculum in the host population by the predator may be beneficial for inoculative releases of microsporidian pathogens. Because no indication of an infection of *C. sycophanta* with the microsporidia of its prey occurred, we further conclude that no severe negative effects arise for the predator.
